# A GPU-Parallel Image Coregistration Algorithm for InSar Processing at the Edge

**DOI:** 10.3390/s21175916

**Published:** 2021-09-02

**Authors:** Diego Romano, Marco Lapegna

**Affiliations:** 1 Institute for High Performance Computing and Networking (ICAR), CNR, 80131 Naples, Italy; 2 Department of Mathematics and Applications, University of Naples Federico II, 80126 Naples, Italy; marco.lapegna@unina.it

**Keywords:** InSAR, remote sensing, onboard processing, cross-correlation, GPU-parallel, computation offloading, edge computing, CUDA

## Abstract

Image Coregistration for InSAR processing is a time-consuming procedure that is usually processed in batch mode. With the availability of low-energy GPU accelerators, processing at the edge is now a promising perspective. Starting from the individuation of the most computationally intensive kernels from existing algorithms, we decomposed the cross-correlation problem from a multilevel point of view, intending to design and implement an efficient GPU-parallel algorithm for multiple settings, including the edge computing one. We analyzed the accuracy and performance of the proposed algorithm—also considering power efficiency—and its applicability to the identified settings. Results show that a significant speedup of InSAR processing is possible by exploiting GPU computing in different scenarios with no loss of accuracy, also enabling onboard processing using SoC hardware.

## 1. Introduction

The InSAR technique requires the alignment of two SAR images (called primary and secondary) of the same area, captured by spatially separated antennas on the same platform or by a single antenna with near-repeat ground-tracks, to measure phase difference for ground-elevation estimation. Possible applications are in topographic mapping, environmental monitoring, and planetary exploration [1]. Coregistration of images is a necessary step to increases the coherence of the interferogram and improves the quality of the phase unwrapping procedure [2]. In the case of space-borne platforms, a multi-temporal acquisition is the most common modality, requiring efficient and precise coregistration of single look complex data [3,4]. Two approaches are common: one based on fringe contrast [5], and the other on spectral cross-correlation [3,6]. With modern computers growing in computational power, the second approach has become preferential in many applications since its applicability in automatic coregistration. A common algorithm consists of using spectral cross-correlation on corresponding small patches of the two equally subdivided images, producing pixel offsets for each pair, followed by a fitting function (e.g., Least Mean Square) to obtain coefficients of the transformation equations [7]. This algorithm can be applied on complex (interferometric phase information) or magnitude-only data, named respectively coherent or incoherent cross-correlation [8]. Both present issues: coherent cross-correlation requires preliminary removal of systematic (non-noise) phase differences, while incoherent cross-correlation needs the oversampling by a factor of two in range direction [9].

On the contrary of optical images, a coregistration with pixel-level accuracy is not adequate for InSAR processing on space-borne platforms, as the resolution of satellite images is not better than a few meters [10]. The phase coregistration must have higher accuracy to obtain a high-quality interference fringe pattern; therefore, a sub-pixel level processing is necessary. Roughly one-tenth pixel is a widely accepted accuracy value, but in literature, some authors consider up-scaling to one-hundredth pixel [11]. One of the most used approaches in spectral cross-correlation consists of two steps: a coarse cross-correlation with pixel-level accuracy to search for coarse image offsets used in secondary image shifting; a fine co-registration with sub-pixel accuracy, to search for sub-pixel tie points used for fitting transformation equations. The second step is usually implemented by up-sampling the area of the coarse cross-correlation peaks and looking for sub-pixel peaks [3] or by over-sampling the patches from the two images and looking for the optimal offset to maximize the cross-correlation of the pairs [12]. Both approaches show promising results, and they mainly differ in computational effort and memory footprint.

During the last ten years, a new envisioning of distributed architectures has emerged. After the disruptive introduction of Cloud computing, which gave new access to HPC resources for Scientific Computing, we observed a higher growth of available computing resources than connectivity. Since transfer bandwidth can get quickly saturated by the amount of data produced by the sensors, the idea of computation offloading found new spaces in the definition of architectures and paradigms. Fog computing [13] defines a model for the deployment of distributed applications and services residing between end-devices and central servers. Edge computing [14] refers to the technologies enabling the computation at the proximity of data sources. In the literature, authors defined many terms to describe similar concepts, and a recent comprehensive survey of relevant concepts and terms is available in [15]. In our view, as described in [13,14], applications in fog computing run in a multi-layered architecture with dynamic reconfigurations, while in edge computing, specific applications run in fixed logic locations. Therefore, we adopted the term edge for this work when discussing both the remote sensor and the user side.

Thanks to the introduction of edge GPU accelerators [16], opportunities for high-performance applications at the edge are growing in number. In the context of aerospace, in [17] a possible adoption of onboard GPUs to process SAR data is introduced. In [18] the authors explore the idea of using GPUs on Low Earth Orbit (LEO) satellites for several possible applications, putting in evidence the opportunities to process data for Earth imagery, weather observations, and remote sensing directly in space. As shown in [19], LEO satellites can even employ COTS like NVIDIA Tegra K1. The authors of [20] introduce the concept of Orbital Edge Computing, which could lead to orbital systems for visual inference overcoming the limits of streaming downlink architectures. In general, aerospace platforms performing sensing and imagery can produce multi-Gbps of data, but transmission capacity to ground stations limits their acquisition time. Moving processing time onboard could decrease the bandwidth cost for transmitting data and increase the operation time of sensors. All the previous considerations motivate the effort to introduce high-performance edge computing algorithms for remote sensing data.

In this work, we present a GPU-parallel image coregistration algorithm for InSar processing based on incoherent cross-correlation in the frequency domain. Starting from an open-source InSAR processing system widely available to the SAR community, namely GMTSAR [21], we designed an efficient GPU-parallel algorithm that minimizes memory transfers, and by exploiting peculiarities of the chosen problem decomposition to fit in several hardware configurations, minimizes algorithmic overhead [22].

We used CUDA architecture [23] to implement the presented GPU-parallel algorithm on four different NVIDIA GPUs introducing several scenarios. We compared the results with the reference sequential GMTSAR implementation and another GPU implementation in the literature that exploits OpenCL and follows the same sequential GMTSAR algorithm [24]. We validated the quality of the proposed algorithm through such comparisons, showing excellent accuracy for several sensors and significant improvements in performance.

This work contributes to evince the opportunity to use GPU on edge resources for onboard InSAR processing by designing an efficient GPU-parallel algorithm for image coregistration. By moving the processing onboard, sensor uptime can increase while reducing downlink bandwidth usage. Nevertheless, thanks to a dynamically configurable problem decomposition, our GPU-parallel algorithm can process data in two additional settings: near the user on her workstation or in the cloud on HPC resources. A detailed performance analysis shows that our GMTSAR component for cross-correlation on GPU can contribute to the InSAR scientific community by speeding up the analysis of SAR images from several missions.

The paper has the following organization: in Section 2, we present the preliminary design approaches and the detailed parallelization steps to implement the GPU-parallel algorithm for cross-correlation; in Section 3, we analyze the results of our CUDA implementation from several points of view, namely accuracy, parallel performance, and energy efficiency; finally, in Section 4 we discuss the outcome of our research, composing four possible application settings.

## 2. Towards a GPU-Parallel Algorithm

As previously introduced, the design of our GPU-parallel algorithm starts from the analysis of GMTSAR, an open-source InSAR Processing System rich in tools for interferometry on data from several SAR sensors and missions. In GMTSAR, image coregistration processes two Single Look Complex (SLC) files previously obtained by proper focusing of raw data. The algorithm splits into two codes:

- *xcorr*, a C program implementing incoherent spectral cross-correlation (Actually, *xcorr* implements also time correlation, but to the best of our knowledge, no available script uses this feature as main coregistration tool), which computes local sub-pixel offsets for each pair of corresponding patches within the SLC images;
- *fitoffset.csh*, a C shell script implementing least-square fitting of local offsets through *trend2d* tool from GMT [25], producing the transforming coefficients.

The most significant computational load of GMTSAR coregistration lies in the cross-correlation step, which is also the most promising for effective problem decomposition. We will focus on this part and develop a parallel strategy starting from the pseudo-code description of sequential Algorithm 1.

**Figure 1:**
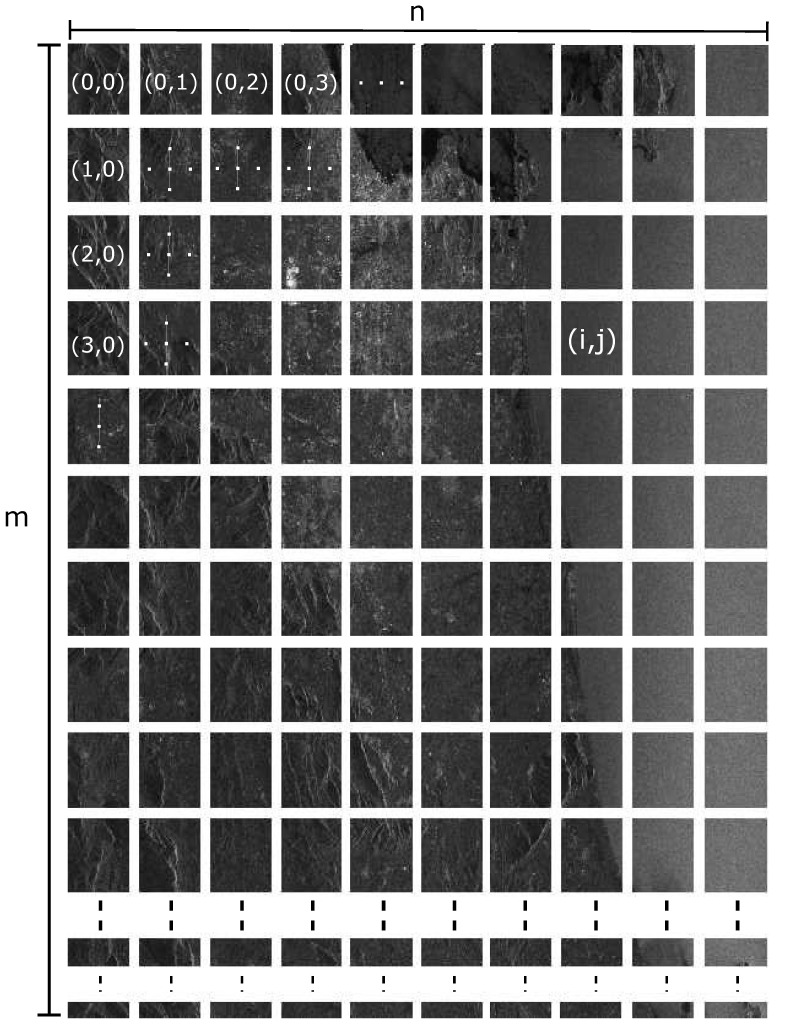
Partitioning of a SLC image into patches.

The main body of *xcorr* consists in an initial equal partitioning of the two SLC images, as in Figure 1, applying an initial offset on the secondary image if this is the case, followed by two nested cycles to cross-correlate each patch in the primary image with the corresponding patch in the secondary image.

Preliminarily, interpolation on range direction and conversion in amplitude, with mean subtraction, precede the patch correlation steps, as we can classify the algorithm as an incoherent cross-correlation. Then, a frequency correlation with pixel accuracy follows, as described in function do_freq_correlation. Finally, in do_highres_correlation, a subset of correlation data is interpolated to obtain sub-pixel accuracy.

### 2.1. Decomposition

The first step in designing an efficient GPU-parallel algorithm is to decompose the computational problem into smaller sub-problems, which are eventually solved in parallel [26]. In order to correctly identify the most suitable decomposition in the context of the application, a mapping of possible decompositions onto the available computational resources is a fundamental step to ensure high efficiency in the resulting algorithm.

Looking at the image subdivision in the sequential Algorithm 1, and considering a MIMD computing architecture $M_{P}$ with *P* processing units, natural coarse-grain decomposition of the problem consists in assigning to each unit the computation of the offset for a single pair of patches, both at pixel and sub-pixel level.

If we denote with (i,j) the same 2D integer indexing for the partitioning on $I_{p}$ and $I_{s}$, where 0⩽i<m in azimuth and 0⩽j<n in range direction, and if m·n⩽P, we can represent the decomposition as:

$$d_{1}:\left(C_{p}\left(i,j\right),C_{s}\left(i,j\right)\right)\to p\left(i,j\right)$$

where $p\left(i,j\right)\in M_{P}$. In this optimistic hypothesis, each p(i,j) can execute on a different patch pair –concurrently with other units– the operations described on lines 6–9 in Algorithm 1. The gain in terms of execution time would be the maximum for the chosen partitioning, i.e., speed-up $S_{p}\approx m\cdot n$.

Realistically, m·n>P and $p\left(i,j\right)=p\left(i^{\prime },j^{\prime }\right)$ for some $\left(i,j\right),\left(i^{\prime },j^{\prime }\right)$ pairs. Hence, if *P* is divisor of m·n then $S_{p}\approx P$, which is optimal with respect to $M_{P}$.

Note that most of the computations consist of direct numerical methods (FFT, Hadamard products, normalized time correlation, searching an unsorted array). By fixing the dimensions of all patches to size k×l, with k=M/m and l=N/n, we can perform the computations mentioned above on a SIMD architecture, i.e., that can execute the same operations synchronously on several data elements from patches containing the same quantity of data. We can set this hypothesis as true in the *xcorr* method. Since GPUs use a SIMT execution model and admit a little divergence, we can adopt decomposition $d_{1}$ at a high level, where p(i,j) are mapped onto the Streaming Multiprocessors (SM) units [22]. Usually, the number of SMs available on a GPU is much less than m·n, and it can only be incidentally a divisor of the total number of patches.
**Algorithm 1:** GMTSAR *xcorr* algorithm. 
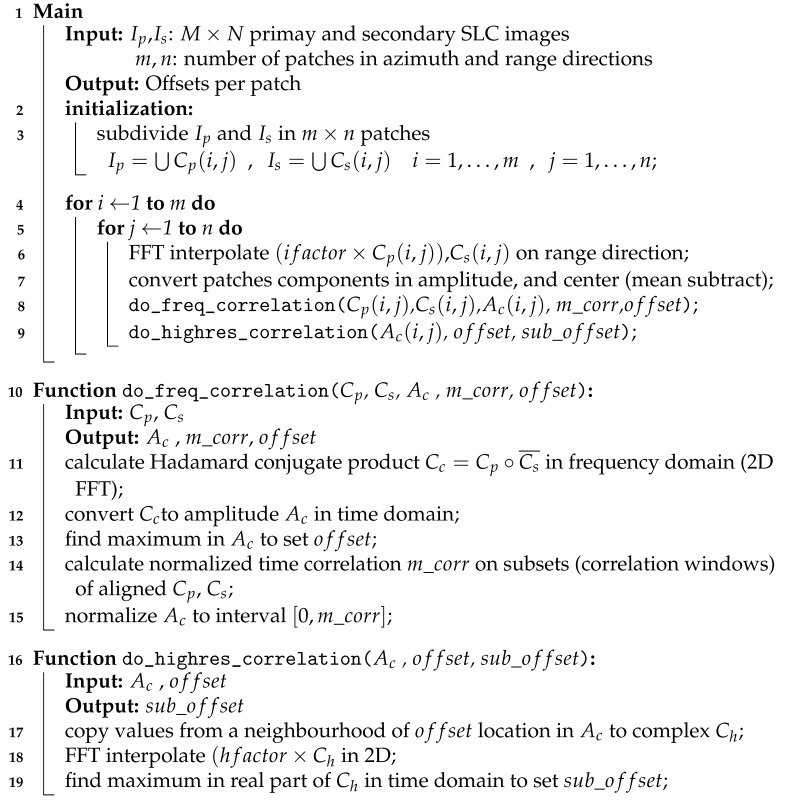



Each SM is provided with several Streaming Processors (SP, also called CUDA cores on NVIDIA architectures), exposing a possible low-level (fine-grain) decomposition:

$$d_{2}\left(i,j\right):\left(E_{p}\left(u,v\right),E_{s}\left(u,v\right)\right)\to t\left(u,v\right)$$

where, for each patch pair $\left(C_{p}\left(i,j\right),C_{s}\left(i,j\right)\right)$ with index (i,j), we can assign the operations needed for a pair of elements $\left(E_{p}\left(u,v\right),E_{s}\left(u,v\right)\right)$ to an execution thread t(u,v), with $E_{p}\left(u,v\right)\in C_{p}\left(i,j\right)$, $E_{s}\left(u,v\right)\in C_{s}\left(i,j\right)$, and 0⩽u<k, 0⩽v<l. In other words, each thread works on a single piece of data. On GPUs, we can map threads t(u,v) onto the SPs through the GPU execution configuration in terms of threads organized in blocks within a grid.

### 2.2. Data Structures

In the general context of high-performance parallel computing, one of the primary overhead sources lies in memory access to data structures (e.g., [27]). Such overhead is an even more critical problem in GPU computing, where data reside both on the host (motherboard with CPU) and the device (GPU). An accurate design of arrays and other structures is essential to deliver efficient software.

Let analyze the memory accesses requested by the computational kernels in *xcorr*. The kernel with the highest time complexity is the Fast Fourier Transform, which for NVIDIA GPUs is available on the library CUFFT. Its main component is efficient when used on batches of arrays of length equal to a power of 2, as it better exploits the underlying algorithm on the employed parallel architecture. In our case, we can use this hypothesis on the array length since it is easily achieved by setting proper patch dimension or by zero padding.

Line 6 of Algorithm 1 requires the execution of an FFT batch of 1D-arrays along range direction, for each $C_{p}\left(i,j\right)$ and $C_{s}\left(i,j\right)$. In CUFFT, if a 1D-array represents a matrix (e.g., in row-major order), by setting a proper stride in memory addressing, the FFTs for every row of the matrix can be efficiently calculated concurrently within a parallel batch. In the context of our algorithm, a single FFT batch run can transform all the range lines in a patch.

**Figure 2:**
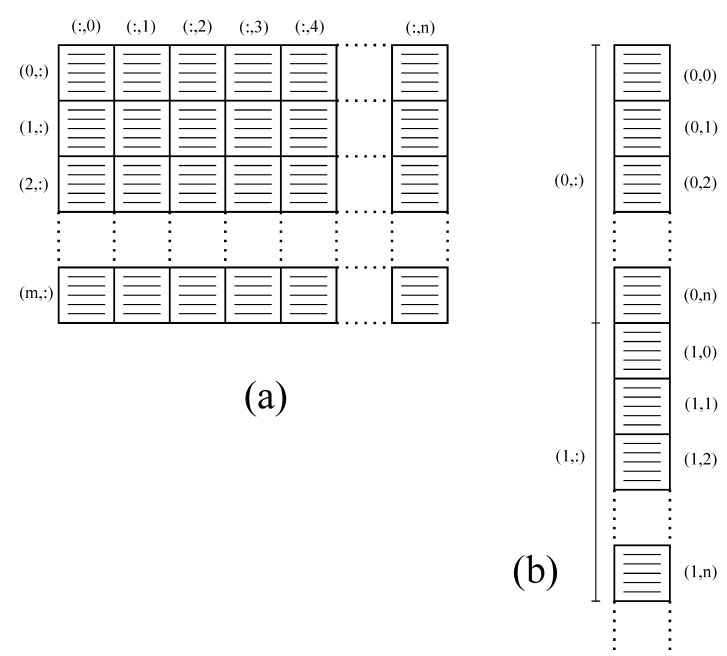
Reorganization of an input image (**a**) as a strip of patches (**b**).

Decomposition $d_{1}$ assigns each patch pair to an SM, allowing the processing of several patches concurrently on the GPU. Therefore, we can configure an FFT batch to process range lines taken from several patches to be executed by CUFFT with the best performance on the available hardware. This batch requires a reorganization of input images, typically formatted in M×N matrices arranged in row-major 1D-arrays, as strips of patches in (m·n·k)×l row-major 1D-arrays, as depicted in Figure 2. The new array can be formatted during memory copy from host to device, concatenating as many patches as the available device memory.

At this point, we can introduce a new low level decomposition, similar to $d_{2}$, but with a different indexing:

$$d_{3}:\left(E_{p}\left(r,s\right),E_{s}\left(r,s\right)\right)\to t\left(r,s\right)$$

where we can assign the operations needed for a pair of elements $\left(E_{p}\left(r,s\right),E_{s}\left(r,s\right)\right)$ in the strip of patches, to an execution thread t(r,s), without considering patch indexing.

Hence, we can easily use the above array structure also for the computations on lines 7, 11, 14 of Algorithm 1 and storing the resulting $A_{c}\left(i,j\right)$ and consequential macro-operations on lines 12, 13, 15, 17. Some additional difficulties arise when transforming $C_{h}\left(i,j\right)$ on line 18, also memorized as a strip of patches, because FFT interpolation in 2D requires two batches: one in the range direction (as on line 6) and one in the azimuth direction. For the latter, the batching mechanism of CUFFT, which does not directly support column-wise batches, requires two further matrix transpositions.

### 2.3. GPU Kernels

We present a sketch of the main procedure from the GPU-parallel algorithm in Algorithm 2. Using decomposition $d_{1}$ and the data structures described above, we can assign to the GPU the operations within the for cycle.
**Algorithm 2:** GPU-parallel main algorithm. 
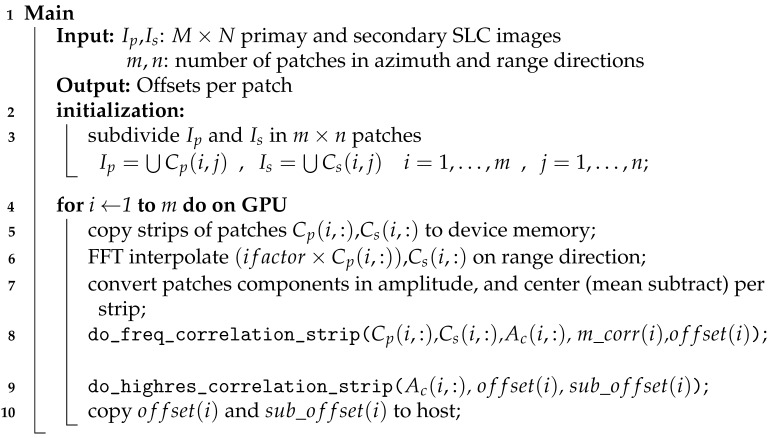



**Figure 3:**
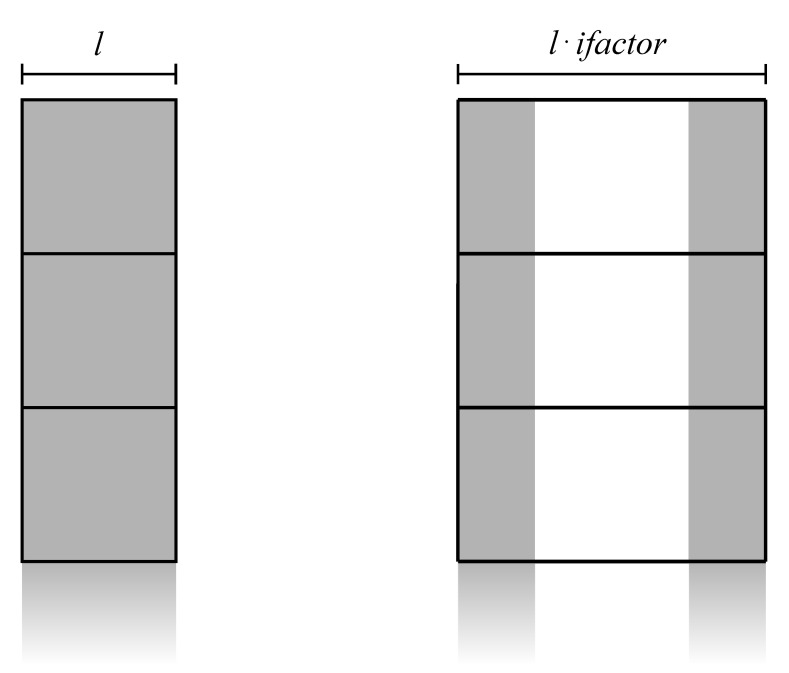
Splitting of input data into two vertical blocks copied on a zeroed larger array. ifactor is the interpolation factor.

**Figure 4:**
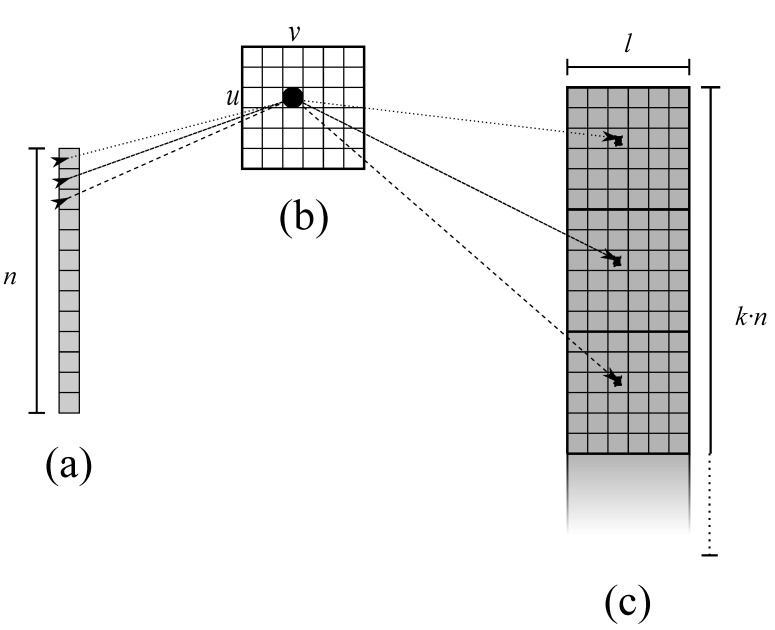
Graphical representation of $d_{2}$ configuration for patch centering on line 7 of Algorithm 2. (**a**) is the vector with mean values per patch, stored in a fast shared memory; (**b**) are threads t(u,v), one per patch element; (**c**) is the strip of patches to be centered by subtracting corresponding mean values.

Let us create patch strips concatenating one patch row per strip, using data copy from host to device memory for data formatting described in Section 2.2. The next steps are detailed as follows:

**Line** **6** The first macro-operation on GPU is the FFT interpolation on range direction. It consists in:
  6.1 preliminary transformation in frequency domain along rows, exploiting CUFFT;
  6.2 zero-padding in the middle of each row to extend its length by ifactor (e.g., 2× the original length);
  6.3 transformation back in time domain, also implemented with CUFFT;
  6.4 final point-wise scaling to compensate the amplitude loss induced by interpolation (see [28] for more details).
  With the idea of taking advantage of both high bandwidth memory and its CUDA advanced copying tools, we can implement zero padding by splitting the input data into two vertical blocks copied on a zeroed larger workspace array (see Figure 3). Then, we can efficiently implement the final point-wise scaling using low-level decomposition $d_{3}$ on the entire strip length. The device memory footprint for completing the first step, supposing to execute FFT in place, is (k·n)×(l·ifactor).
**Line** **7** The following step consist in three sub-steps:
  7.1 point-wise conversion to amplitude, decomposed by $d_{3}$;
  7.2 computation of the mean value per patch, decomposed by $d_{1}$;
  7.3 point-wise subtraction per patch, decomposed by a modified $d_{2}$.
  The first conversion can be designed as the previously described scaling kernel, copying result in a work area for the following sub-step. Then, by adopting a good strategy for GPU-parallel reduction available in the CUDA Samples, we can conveniently split the k·n rows of the strip into a number of threads divisor of *n*, and compute each mean value per patch. For this operation, an additional array of *n* elements is necessary to store the results. Finally, a point-wise subtraction is necessary to implement patch centering, subtracting the same mean value from every point in the same patch. Thus, to optimize memory accesses, decomposition $d_{2}$ will be configured to assign every $E_{p}\left(u,v\right)\in C_{p}\left(i,j\right),\forall j<n$ to the same t(u,v). In other words, each t(u,v) will compute *n* subtractions accessing all *n* mean values stored in a fast shared memory (see Figure 4). The same will be applied to $E_{s}\left(u,v\right)$. The additional memory footprint for this step is *n* locations to store mean values.

Algorithm 3 presents the procedure to process on GPU each pair $\left(C_{p},C_{s}\right)$ of patch strips to obtain coarse offsets (at pixel level) from spectral cross-correlation. A secondary output, needed for the following high-resolution correlation at sub-pixel accuracy, is patch strip $A_{c}$ containing normalized values of correlation matrices.

Steps in Algorithm 3 are detailed as follows:
**Algorithm 3:** GPU-parallel algorithm for frequency cross-correlation. 
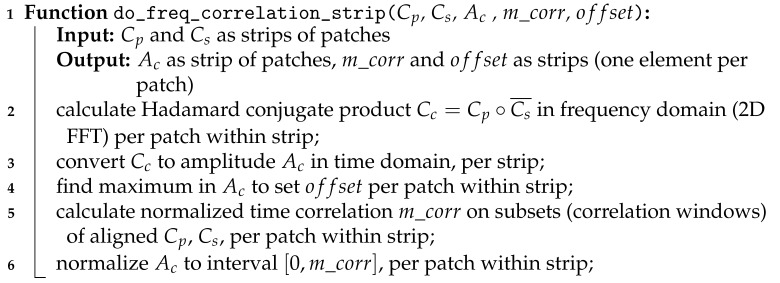

**Line** **2** The calculation of Hadamard conjugate product $C_{c}=C_{p}\circ \bar{C_{s}}$ in frequency domain consists in:
  2.1 preliminary transformation in frequency domain of both $C_{p}$ and $C_{s}$, per patch, exploiting 2D CUFFT;
  2.2 point-wise conjugate product;
  2.3 transformation back in time domain of resulting $C_{c}$, also implemented with 2D CUFFT.
  We can configure the 2D FFTs straightforwardly using the data structure from Section 2.2 for strip-aligned patches. Then, we can decompose the point-wise product using $d_{3}$, including computation of local complex conjugate. The additional memory footprint needed for this step, supposing to execute FFT in place, corresponds to the storage memory for $C_{c}$ which is (k·n)×l.
**Line** **3** We can implement the conversion as Line 7.1 in Algorithm 2.
**Line** **4** This reduction step can be decomposed and implemented as Line 7.2 in Algorithm 2.
**Line** **5** This step consists in computing the normalized cross-correlation in time domain:
  $$m\_corr\left(i,j\right)=\frac{\sum_{u}\sum_{v}E_{p}\left(u,v\right)\cdot E_{s}\left(u,v\right)}{\sqrt{\sum_{u}\sum_{v}E_{p}{\left(u,v\right)}^{2}\cdot \sum_{u}\sum_{v}E_{s}{\left(u,v\right)}^{2}}}$$
  where $C_{p}\left(i,j\right)$ is aligned to $C_{s}\left(i,j\right)$ by offset(i,j), $C_{p}^{c}\subset C_{p}$ and $C_{s}^{c}\subset C_{s}$ are the correlation windows, and $E_{p}\left(u,v\right)\in C_{p}^{c}\left(i,j\right)$, $E_{s}\left(u,v\right)\in C_{s}^{c}\left(i,j\right)$ with $0⩽u<k_{c}<k$, $0⩽v<l_{c}<l$.
  More in details:
  5.1 align $C_{p}\left(i,j\right)$ and $C_{s}\left(i,j\right)$, per patch within strip, and store correlation windows in $C_{p}^{c}\left(i,j\right)$ and $C_{s}^{c}\left(i,j\right)$;
  5.2 point-wise products of
$c\left(u,v\right)=E_{p}\left(u,v\right)\cdot E_{s}\left(u,v\right)$
$a\left(u,v\right)=E_{p}\left(u,v\right)\cdot E_{p}\left(u,v\right)$
$b\left(u,v\right)=E_{s}\left(u,v\right)\cdot E_{s}\left(u,v\right)$
    per strip;
  5.3 computation of the three sums, per patch within strip;
  5.4 computation of m_corr(i,j) per patch within strip.
  The alignment in sub-step 5.1 can be implemented through data copy from host to device with proper offset. Since products in 5.2 are similar to point-wise operators in Line 3, we can use the same decomposition $d_{3}$. The sum is a reduction, and sub-step 5.3 can use the same decomposition as Line 4. Finally, sub-step 5.4 consists of n≈20 products, and we can implement them sequentially. The additional memory footprint for this step corresponds to the two arrays for the strips of aligned patches, which consists of $\left(k_{c}\cdot n\right)\times l_{c}$ elements each, where $k_{c}\times l_{c}$ is the size of the correlation window.
**Line** **6** The normalization can follow the same strategy as Line 7.3 in Algorithm 2.

In Algorithm 4, we can find the steps to compute sub-pixel offsets.
**Algorithm 4:** GPU-parallel algorithm for high-resolution correlation. 
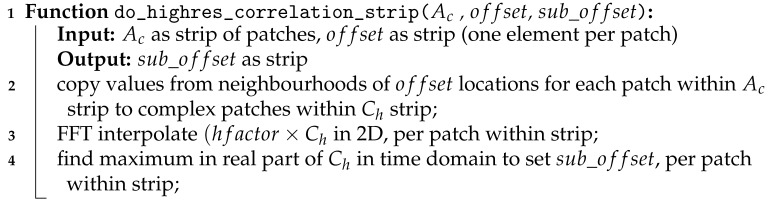



In details:

**Line** **2** We can implement the copy of correlation values from $A_{c}$ to $C_{h}$ adopting $d_{3}$, assigning the floating-point values to the real part of the complex array. This step is necessary to reorganize data for FFT interpolation in 2D. We choose data from $A_{c}$ in neighborhoods of pixel-level offsets from Algorithm 3 and then incorporate them in an array with the same patch strip structure as described in Section 2.2. Additional memory footprint for this step correspond to the size of a complex array with $\left(k_{h}\cdot n\right)\times l_{h}$ elements, where $k_{h}\times l_{h}$ are the dimension of the neighbourhood around each offset per patch.
**Line** **3** This step can be implemented similarly to Line 6 of Algorithm 2, but with additional matrix transpose:
  3.1 preliminary transformation in frequency domain along row, exploiting CUFFT;
  3.2 zero-padding in the middle of each row to extend its length by hfactor (e.g., 16× the original length);
  3.3 transformation back in time domain, also implemented with CUFFT;
  3.4 matrix transpose, to arrange data for the subsequent processing along columns of $C_{h}$;
  3.5 as 3.1;
  3.6 as 3.2;
  3.7 as 3.3;
  3.8 matrix transpose, to re-arrange data back to the original ordering.
  The newly introduced matrix transpose follows a good GPU-parallel strategy available in the CUDA Samples involving shared memory tiles. We can imagine the strip of patches as a single matrix with $k_{h}\cdot n$ rows. The additional memory footprint for this step consist in three temporary strips of patches: the first to interpolate along rows with $\left(k_{h}\cdot n\right)\times \left(l_{h}\cdot hfactor\right)$ elements; the second, with $\left(l_{h}\cdot hfactor\right)\times \left(k_{h}\cdot n\right)$ elements, to transpose the matrix; the third to interpolate along the other direction with $\left(l_{h}\cdot hfactor\right)\times \left(\left(k_{h}\cdot hfactor\right)\cdot n\right)$ elements. Final 2D interpolated data will need another array of $\left(\left(k_{h}\cdot hfactor\right)\cdot n\right)\times \left(l_{h}\cdot hfactor\right)$ complex elements.
**Line** **4** This reduction step can be implemented as Line 4 of Algorithm 3, working on the real part of the array $C_{h}$.

## 3. CUDA Implementation: Results

This section presents the accuracy, parallel performance, and energy efficiency of the GPU-parallel cross-correlation algorithm implemented in CUDA. We introduce four different hardware configurations, which we consider significant for the following three scenarios:

- Edge Computing near the sensor, with a System-on-Chip usable onboard a SAR platform;
- Edge Computing near the user, with two different workstation configurations;
- Component for a Cloud Computing application, with a typical GPU Computing configuration.

The hardware configurations for the three cases are as follows:

**Jetson** **Nano** This is a small computing board (69×45 mm) consisting of a stripped-down version of Tegra X1 (System-on-Chip). It integrates an NVIDIA Maxwell GPU with 128 CUDA cores on one Multiprocessor running at 921 MHz, sharing a 4 GB 64-bit LPDDR4 memory chip with a 4-core CPU ARM Cortex A57 running at 1.43 GHz. We used a development kit configuration for testing, which has a slot for an additional microSD card containing OS Ubuntu 18.04 LTS, software, and data;
**Q** **RTX** **6000** A workstation configuration: a graphic card NVIDIA Quadro RTX 6000 with 4608 CUDA cores on 72 Multiprocessors running at 1.77 GHz with a global memory of 22 GBytes, and 10-core CPU Intel Xeon Gold 5215 running at 2.5 GHz, OS CentOS Linux 7 (Core);
**GTX** **1050** **Ti** Another workstation configuration: a graphic card NVIDIA GeForce GTX 1050 Ti with 768 CUDA cores on 6 Multiprocessors running at 1.42 GHz with a global memory of 4 GBytes, and 4-core CPU Intel Core i7-7700 running at 3.6 GHz, OS Linux Ubuntu 20.04.2 LTS;
**Tesla** **V100** This is our data center configuration: the single node has an NVIDIA Tesla V100-SXM2-32GB with 5120 CUDA cores on 80 Multiprocessors running at 1.53 GHz with a global memory of 32GBytes, and a 16-core CPU Intel Xeon Gold 5218 running at 2.30GHz, OS CentOS Linux 7 (Core).

Using data from GMTSAR samples [29], we tested our GPU-parallel algorithm on ALOS, ALOS-2, Envisat, ERS, COSMO-SkyMed, RADARSAT-2, and TerraSAR-X images.

### 3.1. Accuracy

We compare the output of our *xcorr-gpu* with the original *xcorr*, using the same input files and arguments, and we evaluate differences in calculated offsets both per patch and globally, i.e., after the final fitting step.

Following the correspondent shell script to process each dataset, the arguments for the subdivisions are as reported in Table 1.

The output of *xcorr* consists of five columns, reporting on each line per patch: *x* and *y* coordinates of the central pixel in the primary image, estimated *x* and *y* offsets, and calculated correlation (normalized in the time domain). We consider this output as the reference to calculate errors introduced by our GPU-parallel version.

As each column represent a vector, to fairly approach the comparison we need to measure the size or norm of it. Let us call *x* a column vector, we consider

$${∥x∥}_{2}=\sqrt{\sum_{i}{|x_{i}|}^{2}}\;,\;{∥x∥}_{\infty }=\max_{i}|x_{i}|$$

respectively the two-norm and the infinity-norm of *x*. If $\hat{x}$ is an approximation calculated by *xcorr-gpu*, we will refer to

$$\eta_{p}=\frac{{∥\hat{x}-x∥}_{p}}{{∥x∥}_{p}}$$

as the relative error in $\hat{x}$ using *p*-norm, where *p* is 2 or *∞*, and assuming ${∥x∥}_{p}\neq 0$.

In Table 2, we report measured relative errors, using both two-norm and infinity-norm. For *y* offset, in half cases, we obtained exactly the same values as from the original software. The other cases are accurate to 4 decimal digits or more. A similar situation is for correlation values, where ALOS-1 (ERSDAC format) and ERS present not errors, while for the other datasets, we have the accuracy to 4 decimal digits or more. About *x* offset, three dataset present no error, and the other cases are accurate to 3 decimal digits or more, except for ALOS-2 L1.1 ScanSAR which presents a $\eta_{\infty }=0.130407286$ and $\eta_{2}=0.025773552$. $\eta_{\infty }$ represents the maximum error per vector component, i.e., on 4096 correlated patches, some have a relative error of such magnitude, but we have no information on how it is related to the final coregistration output. $\eta_{2}$ suggests that the error is not present in many components, but we must evaluate the final fitting output to have an insight into such inaccuracy.

**Figure 5:**
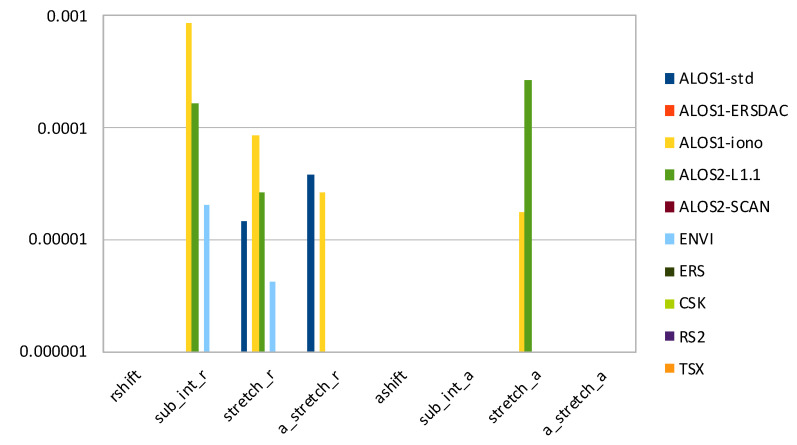
Histogram of relative error (in logarithmic scale) on output values from *fitoffset.csh*, when cross-correlation is calculated with *xcorr-gpu*.

In Figure 5, we can visualize the magnitude of relative error on the output least square fitting implemented by *fitoffset.csh* in the GMTSAR package, when cross-correlation is calculated using *xcorr-gpu*, with respect to the original *xcorr* software. The eight values in the output are:

- rshift—range shift to align secondary image to primary image (in pixels);
- sub_int_r—decimal part of rshift;
- stretch_r—range stretch versus range;
- a_stretch_r—range stretch versus azimuth;
- ashift—azimuth shift to align secondary image to primary image (in pixels);
- sub_int_a—decimal part of ashift;
- stretch_a—azimuth stretch versus range;
- a_stretch_a—azimuth stretch versus azimuth.

As we can see, co-registration of images for most datasets is identical using the original GMTSAR software or our GPU-parallel version. Even with the ALOS-2 L1.1 ScanSAR dataset, we do not report any differences despite the above reported pointwise inaccuracies: on such a long vector (4096 elements), the least square fitting has canceled the effects of marked relative errors. The highest relative error is on the 4th digit of sub-pixel range shift and azimuth stretch versus range for ALOS-1 L1.0 (ionospheric correction) and ALOS-2 L1.1 datasets.

Investigating the algorithmic steps of the GPU-parallel version, we identified in the FFT execution the source of such minimal accuracy differences. Moreover, changing the version of CUFFT on different hardware architecture, no notable changes got our attention. While changing the FFT library for GMTSAR showed slight differences in the output, but nothing valuable for further discussion.

For completeness, we made the same evaluation using the pre-existent parallel OpenCL software *xcorr2-cl* [24], comparing accuracy with our *xcorr-gpu*. This software uses ArrayFire [30] in the stack for several functions (namely FFT). As shown in Table 3, for three datasets, ALOS-1 L1.0 (ionospheric correction), ALOS-2 L1.1, and TerraSAR-X, we got unusable cross-correlation output running *xcorr2-cl*, with two-norm and infinity-norm relative errors higher than 1, that is greater than the modulus of the expected output. In the latter two cases, *fitoffset.csh* failed to run and produce an output. For this reason, we do not report any figures about those datasets.

**Figure 6:**
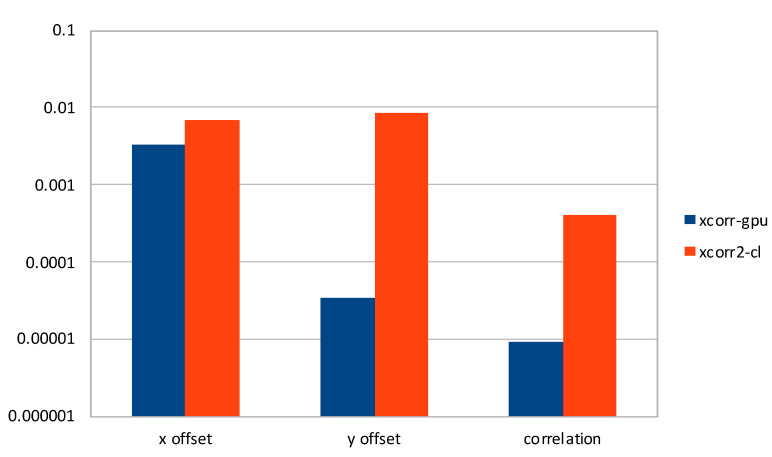
Comparison of average $\eta_{2}$ on selected datasets for *xcorr-gpu* and *xcorr2-cl*, in logarithmic scale.

**Figure 7:**
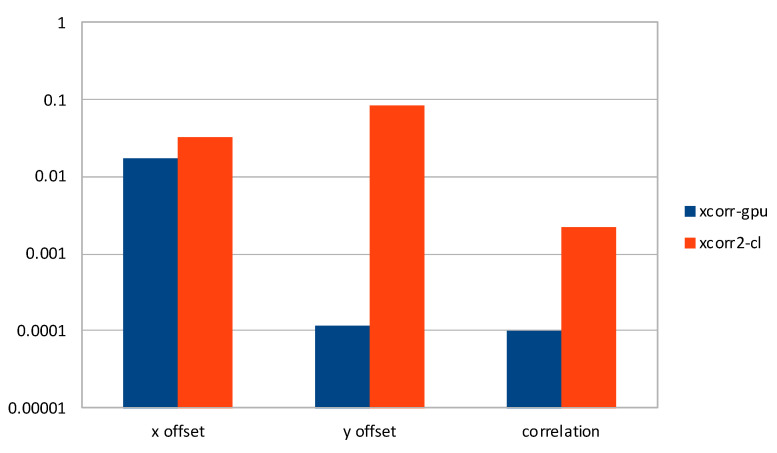
Comparison of average $\eta_{\infty }$ on selected datasets for *xcorr-gpu* and *xcorr2-cl*, in logarithmic scale.

In Figure 6, we present a comparison of average $\eta_{2}$ on *x* and *y* offsets, and calculated correlation, considering all datasets except the three mentioned above, for *xcorr-gpu* and *xcorr2-cl*. We found that our software produces results with better accuracy of two orders of magnitude on two vectors. On the other hand, *x* offsets are accurate to three decimal digits for both pieces of software, with ours having a better accuracy on average. Figure 7 leads to similar reasoning, exacerbating the average errors for *xcorr2-cl* on *y* offsets.

**Figure 8:**
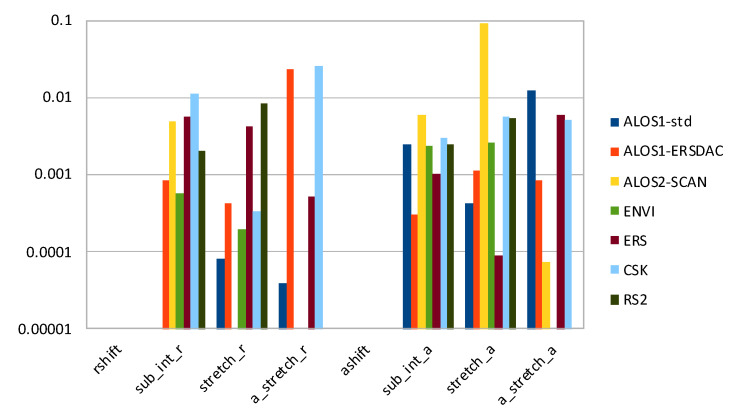
Histogram of relative error (in logarithmic scale) on output values from *fitoffset.csh*, when cross-correlation is calculated with *xcorr2-cl*.

Finally, Figure 8 presents the magnitude of relative error on the output least square fitting implemented by *fitoffset.csh* in the GMTSAR package, when cross-correlation is calculated using *xcorr2-cl*, with respect to the original *xcorr* software. Again, we eliminated the three datasets mentioned above because the output of cross-correlation was unusable. The only parameters estimated exactly are the range and azimuth shifts with pixel precision; all the others present errors with different magnitude per case, in some cases severely impacting on the coregistration.

Looking for sources of inaccuracy in *xcorr-cl*, we analyzed the algorithm as described in [24] and the code available on GitHub repository at https://github.com/cuihaoleo/gmtsaroptimize (Last accessed on 31 August 2021). The algorithmic steps follow those of original *xcorr* in GMTSAR, as outlined in Algorithm 1, with a trivial problem decomposition. Indeed, its GPU parallelism depends on the modules available in ArrayFire, and it is limited to data parallelism within interpolation and correlation steps per patch couples. So probably, the numerical inaccuracy resides in the implementation details. For example, the authors implemented SLC data loading from scratch, resulting in possible misalignments among range lines or overlooking other scaling factors when changing the sensor. From this point of view, we had a conservative approach by untouching pre-existent host-side code for consolidated sequential processing and explicitly implementing data movements to and from device memory. Another example of misalignment is evident in the high-resolution correlation step: the original GMTSAR code had a possible memory violation when building the search window around a peak close to the border. The authors corrected the fault in recent versions, avoiding loading elements with an index less than zero and leaving whatever was already in memory in those window locations. We choose a more neutral approach by zero-padding those memory locations, while the *xcorr-cl* authors choose to move the search window significantly far from the border, with a consequent misaligned peak search.

### 3.2. Parallel Performance

To evaluate parallel performance, we run *xcorr-gpu* on the four hardware configurations reported above and measured total execution time, inclusive of I/O, for ten executions, averaging for realistic figures.

To measure the performance gain of our GPU-parallel algorithm, with respect to the sequential software, we run *xcorr* on the same hardware configurations, except Jetson Nano, where the sequential time would be too long to be of interest in an onboard setting. The average execution time for each dataset is then divided by the average GPU-parallel time to obtain a Speed-Up measure.

Since GMTSAR is based on Generic Mapping Tools [31], the performance of *xcorr* is susceptible to the FFT used. GMT has three options: FFTW (The Fastest Fourier Transform in the West) [32], Kiss FFT [33], and Brenner FFT [34]. The user can choose which version to use or leave it to the automatic decision of GMT. We noticed that on our hardware platforms, GMT automatically chose FFTW, that on the other hand, took the longest execution times. Brenner FFT was the fastest, showing an accuracy equal to FFTW. Kiss FFT, in our testing, was in the mid-range but giving some results with a slightly different accuracy on less than 0.1% of the computed results. We verified that the execution time of *xcorr* for the same dataset with the same FFT algorithm does not significantly change among our different hardware configurations. For a broader perspective, we report the execution times with FFTW and Brenner FFT on GTX 1050 Ti and with Kiss FFT on Tesla V100 and RTX 3070.

In Table 4, we list the execution times of the three *xcorr*, *xcorr-gpu*, *xcorr2-cl* on V100 hardware configuration, with relative speed-ups for our GPU-parallel version and *xcorr2-cl*. For the latter, we removed times and speed-up for three datasets which did not produce a usable results (see Section 3.1). Speed-ups are significant, where our *xcorr-gpu* has an extra average gain of 1.40 with respect to *xcorr2-cl*. We had enough memory to store up to 50 or more patch rows on GPU global memory with this hardware configuration. Such storage enables the total usage of available CUDA cores and generally better performance thanks to data locality on the device. We can think to speed up interferometric analysis of a massive load of data with batch processing on a cluster in this setting.

In Table 5, we list the execution times of the three *xcorr*, *xcorr-gpu*, *xcorr2-cl* on Q RTX 6000 hardware configuration, with relative speed-ups for our GPU-parallel version and *xcorr2-cl*. As before, we removed times and speed-up relative to three datasets for *xcorr2-cl*, which did not produce usable results. Also in this case, speed-ups are significant, where our *xcorr-gpu* has an extra average gain of 1.47 with respect to *xcorr2-cl*. This hardware configuration has enough memory to store up to 50 or more patch rows on GPU global memory, like the V100 case, with all the performance advantages cited above. Nevertheless, we can imagine speeding up interactive interferometric analysis on a high-end workstation when a fast comparison of different acquisitions is the critical point.

On the GTX 1050 Ti hardware configuration, we measured the execution time of *xcorr* using both FFTW and Brenner FFT. Moreover, we isolated the time spent for operations in Supervisor mode. Operations like memory allocation, access to the filesystem, and others that need to run in the Linux kernel space, are counted in this amount. Usually, switching from User to Supervisor mode and back introduce a significant kernel overhead. As shown in Table 6, Brenner FFT spends very little time in Supervisor mode, and the overall execution time is considerably shorter than using FFTW. Hence, a link between more intense usage of Supervision mode and longer execution time seems clear, and it resides in the usage of FFTW that supposedly allocates and deallocates local arrays at the library level, which is transparent to GMT programmers. Brenner FFT is directly encoded in GMT source and probably is optimized for looped and small aligned executions of FFT, as implemented in *xcorr*.

Table 7 presents the execution time on GTX 1050 Ti hardware for *xcorr-gpu* and *xcorr2-cl*, also reporting timings in Supervisor mode. In this case, corresponding to a slightly shorter time in Supervision mode, we observed a longer overall execution time for *xcorr2-cl*. Some source of other proportional overhead is present, supposedly due to the usage of ArrayFire library and to a better definition of multilevel problem decompositions fitting our *xcorr-gpu* algorithm. We notice the enormous difference in terms of speed-up when referencing FFTW or Brenner FFT in the ALOS-2 L1.1 ScanSAR case. The software must operate on 4096 patches for this cross-correlation, amplifying inefficiencies within the original sequential algorithm when using FFTW. In general, speed-ups with respect to Brenner FFT lie in a limited range, e.g., (12.323,28.232) for *xcorr-gpu* and (6.492,18.946) for *xcorr2-cl*, where the first has an extra average gain of 1.64 on the second.

Jetson Nano offers an interesting power management mechanism to optimize power efficiency. Two preset modes of power budget are available:

**5W**  with 2 online CPU cores with a maximal frequency of 918 MHz, and GPU maximal frequency set to 640 MHz;
**10W**  with 4 online CPU cores with a maximal frequency of 1479 MHz and GPU maximal frequency set to 921.6 MHz.

For this hardware configuration, which is relevant in the Edge Computing setting, we do not report the sequential execution time, as it would be too long for our end. At the same time, we cannot compare our GPU-parallel software with *xcorr2-cl*, as Tegra SoC does not support OpenCL, and that software cannot run on such architecture. Table 8 presents the execution times for the different datasets. Since the GPU in the system has only one Streaming Multiprocessor, the total execution time is sensibly longer than those observed on the other hardware configurations. However, it is considerably shorter than the original *xcorr* on commodity processors, and for most cases also when using 5 Watts power mode.

**Figure 9:**
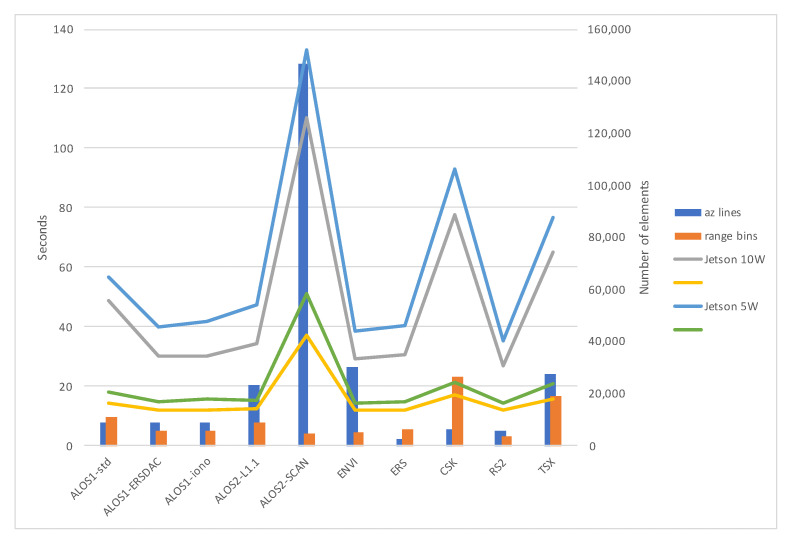
Graphical representation of the execution times for considered datasets on Jetson Nano hardware configuration. Lines represent the values reported in Table 8, bars represent the number of bins in range direction and the number of lines in azimuth direction as reported in Table 1.

An interesting result is a visible link (see Figure 9) between the execution time (and its relative in Supervisor Mode) and the number of bins in the range direction. Even if most of the cross-correlations have the same parameters, and therefore the same work data size, those presenting longer-range lines seem to impact the performance of Jetson Nano indirectly. The number of lines in the azimuth direction does not seem to impact in the same way—the ALOS-2 ScanSAR dataset is processed on 4096 patches instead of 1000 in the other datasets. Analyzing the code execution through the NVIDIA profiler, we found out that memory allocation and copy between host and device introduce a consistent overhead and severely impact performance. From the test, this fact is particularly valid when extracting and aligning patches to build data structures described in Section 2.2. The reason resides in the Jetson Nano memory structure, where both the CPU and the GPU share SoC DRAM memory, each with different accessing and caching behaviors. Having our Jetson Nano CUDA Capability 5.3, pinned memory is not I/O coherent, and its CPU access time is higher because it is not cached on the CPU [35]. Since our code uses pinned memory for better performance on the other hardware configurations, we will consider future portability redesign to improve performance on Jetson boards with lower CUDA Capability.

### 3.3. Energy Efficiency

Thanks to tools available for NVIDIA hardware, we were able to measure the power consumption of our *xcorr-gpu* software on V100 and Jetson Nano configurations. By sampling a milliwatt meter, we estimate the energy consumed by the GPU during the execution of the software.

**Figure 10:**
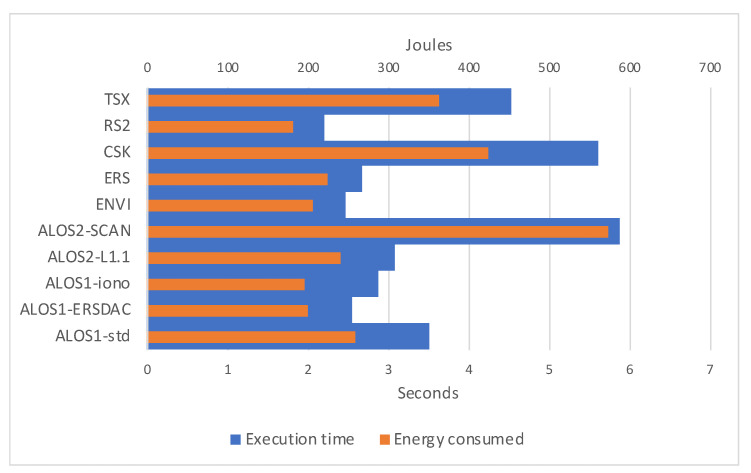
Graphical representation of the execution times for considered datasets on V100 hardware configuration, and relative joules of energy consumed.

In Figure 10, energy consumed per dataset processing superimposes the time taken. Since *Joules = Watte ·seconds*, energy-consuming naturally follows execution time. Some visible differences are due to overhead present in the execution flow. Considering that the TDP (Thermal Design Power) of our V100 is 300 watts, our software stands well behind such limit, offering high-performance rates with limited energy requests.

**Figure 11:**
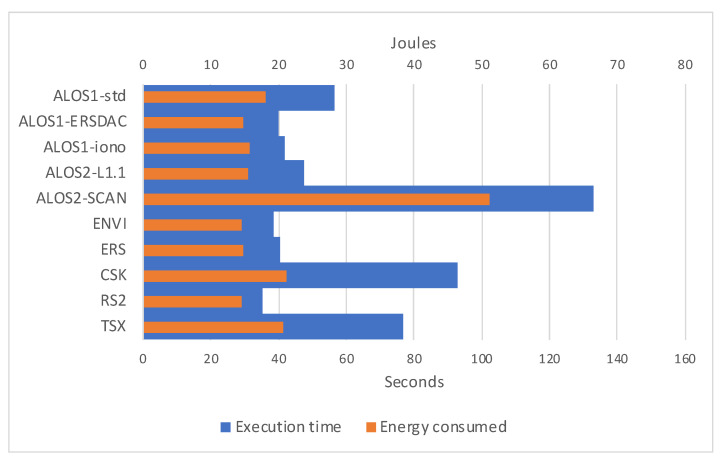
Graphical representation of the execution times for considered datasets on Jetson Nano hardware configuration set on 5-watt power mode, and relative joules of energy consumed.

**Figure 12:**
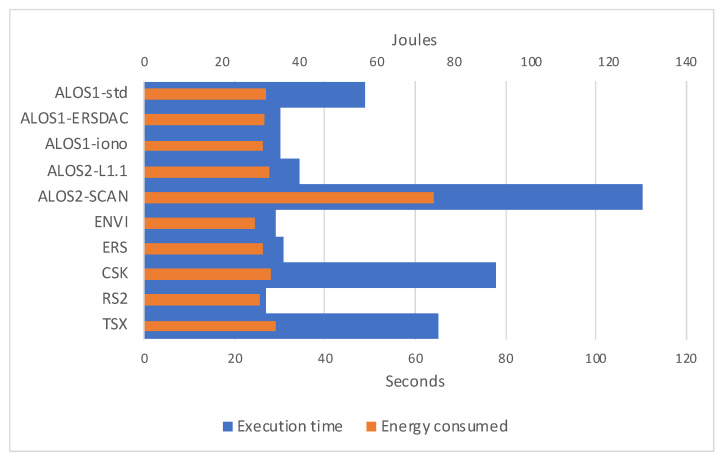
Graphical representation of the execution times for considered datasets on Jetson Nano hardware configuration set on 10-watt power mode, and relative joules of energy consumed.

When analyzing the Jetson Nano case, we need to separate the two cases for 5- and 10-watt power modes, remembering that such watts refer to the entire SoC and not the GPU alone. In Figure 11, we can notice that when running on 5-watt power mode, our software does absorb less than 0.5 watts per second on average. Moreover, energy consumption is more leveled than the V100 case, remarking that longer execution times are mainly due to not energy-consuming overhead. When looking at the 10-watt power mode (Figure 12), energy consumption seems even more leveled, with an average absorption of less than 1 W per second.

## 4. Discussion

Cross-correlation is the most time-consuming kernel when co-registering two SAR images. Such procedure is mainly used during InSAR processing and is currently done offline in ground stations.

Thanks to the consolidation of paradigms like cloud, fog, or edge computing, we can now conceive InSAR processing: near the final user, on workstations provided with modern GPUs; near the sensor, on GPU accelerated SoC; or in the cloud by exploiting hardware resources like HPC clusters provided with special-purpose GPUs for GPGPU.

When processing large datasets with proper references, InSAR coregistration can be easily distributed on several computing units by coupling pairs of images that can be processed concurrently. Currently, GPUs in HPC clusters work as computing accelerators. For this reason, we can speed up the entire process by implementing a proper GPU-parallel algorithm, as we did and tested on the V100 hardware configuration. If the power supply is an issue, we also tested our software for energy consumption, showing that absorption stays well below the TDP of the accelerator even in the most demanding cases. Tests validated the use of our GPU-parallel algorithm as a component for an InSAR cloud computing application.

A typical scenario in the scientific community involves using personal workstations for InSAR workflow, with lengthy processing for interferogram production. For this case, we imagined exploiting the graphic card often already available on the computer to speed up the coregistration step. We tested our GPU-parallel algorithm on two hardware settings typical for workstations and personal computers, one with a professional Quadro RTX 6000 card and the other with a gaming level GTX 1050 Ti. For both cases, we registered important speed-ups that can seriously impact the time-consuming supervised procedure. We can frame this setting as an edge computing case near the user.

As a final perspective, we used our cross-correlation software on an innovative edge computing System-on-Chip, namely the Jetson Nano, to test the opportunity of moving SAR data processing onboard. In this case, we can imagine having several pipelined software processors delivering final results with acceptable latency, providing operational intelligence in real-time. If we consider the acquisition time of data strips and the low energy consumption for this specific processing step, the idea seems reasonable. It could provide an innovative setting for better usage of remote sensors, often underused for critical data transfer to the ground station.

For this work, we analyzed an existing Open Source software, namely GMTSAR, and designed from problem decomposition to CUDA software implementation a GPU-parallel algorithm which demonstrated an accuracy equivalent to the preexisting one on all the available sample datasets. We also compared, both on accuracy and parallel performance, our software to another GPU-parallel component based on OpenCL, designed to substitute the cross-correlating software of GMTSAR and implementing an accelerated version of the original sequential algorithm. Results show that our software delivers correct results in a shorter time than the OpenCL one, which also fails in cross-correlating three different datasets. We observed that the inefficiency of OpenCL software is mainly due to a trivial problem decomposition that did not exploit the numerosity of CUDA cores available on considered GPUs. On the other hand, the reported inaccuracy resides in some misalignments of the search windows with input data. We suspect for both cases that ease of programming with ArrayFire high-level library may have introduced some overlooking. As a final consideration, OpenCL is not available on Jetson Nano, making the OpenCL software unsuitable for the onboard scenario.

As future work, we plan to improve the algorithm to implement it on heterogeneous multicore CPU / GPU architectures, as done in [36], and to optimize the portable design of memory accesses to avoid unwanted overhead on Jetson boards with low CUDA capabilities. Moreover, thanks to the introduction of Volta architecture on the recent Nvidia Tegra series, the availability of tensor cores opens up to new algorithmic designs [37]. Those devices deliver half-precision GEMM (General Matrix Multiply) in one clock cycle, consuming low-energy in edge context. We plan to exploit such characteristics for also other onboard processing of sensor data.

## Figures and Tables

**Table 1:** Datasets from the GMTSAR repository used for testing. For each dataset: the number of patches along range direction and along azimuth; search dimensions in elements on x and y direction, per patch; the number of bins in range direction and the number of lines along azimuth, in the SLC files to correlate.

Dataset	Rng Ptchs	Az Ptchs	Xsearch	Ysearch	Range Bins	Az Lines
ALOS-1 L1.0 (std format CEOS)	20	50	128	128	11,304	9216
ALOS-1 L1.0 (ERSDAC format)	20	50	128	128	5652	9216
ALOS-1 L1.0 (ionospheric correct.)	20	50	128	128	5652	9216
ALOS-2 L1.1	20	50	128	128	9072	23,264
ALOS-2 L1.1 ScanSAR	32	128	32	256	4668	146,512
Envisat	20	50	128	128	5191	30,316
ERS	20	50	128	128	6144	2800
COSMOS-SkyMed	20	50	128	128	26,400	6400
RADARSAT-2	20	50	128	128	3416	5744
TerraSAR-X	20	50	128	128	18,880	27,748

**Table 2:** Accuracy of *xcorr-gpu*. Vectorial relative error $\times 10^{-4}$ for *x* and *y* offsets, and correlation with both two-norm and infinity-norm.

$\times 10^{-4}$	$\eta_{2}\left(x\right)$	$\eta_{\infty }\left(x\right)$	$\eta_{2}\left(y\right)$	$\eta_{\infty }\left(y\right)$	$\eta_{2}\left(\mathrm{corr}\right)$	$\eta_{\infty }\left(\mathrm{corr}\right)$
ALOS1-std	3.6336	78.7273	0.0000	0.0000	0.1302	1.2789
ALOS1-ERSDAC	0.0000	0.0000	0.0000	0.0000	0.0000	0.0000
ALOS1-iono	1.7430	4.8203	0.1606	2.4173	0.0661	1.0841
ALOS2-L1.1	0.2232	6.8359	0.0617	1.9505	0.1113	1.4019
ALOS2-SCAN	257.7355	1304.0729	0.4136	2.0613	0.0674	1.1977
ENVI	0.9211	6.3563	0.0000	0.0000	0.1456	1.1952
ERS	0.0000	0.0000	0.0000	0.0000	0.0000	0.0000
CSK	0.0000	0.0000	0.0000	0.0000	0.1918	1.5716
RS2	1.1243	5.6046	2.1927	4.8602	0.0721	1.3222
TSX	0.1877	2.7850	0.1501	2.4639	0.1526	1.1884

**Table 3:** Accuracy of *xcorr-cl*. Vectorial relative error $\times 10^{-4}$ for *x* and *y* offsets, and correlation with both two-norm and infinity-norm.

$\times 10^{-4}$	$\eta_{2}\left(x\right)$	$\eta_{\infty }\left(x\right)$	$\eta_{2}\left(y\right)$	$\eta_{\infty }\left(y\right)$	$\eta_{2}\left(\mathrm{corr}\right)$	$\eta_{\infty }\left(\mathrm{corr}\right)$
ALOS1-std	11.0006	95.7281	232.7862	2920.3151	6.0454	48.5996
ALOS1-ERSDAC	8.1439	55.1633	10.5945	20.0301	1.7251	8.8869
ALOS1-iono	12,130.5023	9285.8254	520.7860	4685.8129	382.4748	4410.2342
ALOS2-L1.1	8952.9820	13,965.4362	2455.0701	4007.3684	8722.5570	9318.6598
ALOS2-SCAN	350.1252	1363.5214	92.0581	480.5890	2.6375	45.5144
ENVI	46.2160	310.2654	129.1276	488.1701	16.3460	56.1731
ERS	9.0660	35.9363	1.0588	9.2563	2.1657	6.9196
CSK	11.1924	145.2376	5.8288	78.5701	1.7991	1.5716
RS2	20.7378	90.3963	29.0177	38.5728	0.7959	1.3222
TSX	8340.4977	10,780.5088	6546.1723	9227.6133	9145.9832	9347.5936

**Table 4:** Execution times and Speed-Up for *xcorr*, *xcorr-gpu*, *xcorr2-cl* on V100 hardware configuration.

V100	xcorr (KISS)	xcorr-gpu		xcorr2-cl	
	Secs	Secs	Speed-Up	Secs	Speed-Up
ALOS1-std	135.069	3.511	38.470	4.149	32.555
ALOS1-ERSDAC	134.997	2.537	53.211	3.644	37.046
ALOS1-iono	157.726	2.874	54.880		
ALOS2-L1.1	134.679	3.083	43.684		
ALOS2-SCAN	256.437	5.874	43.656	9.078	28.248
ENVI	134.073	2.469	54.303	3.361	39.891
ERS	135.272	2.668	50.702	3.786	35.730
CSK	138.973	5.601	24.812	6.394	21.735
RS2	136.484	2.194	62.208	3.714	36.749
TSX	136.579	4.517	30.237		

**Table 5:** Execution times and Speed-Up for *xcorr*, *xcorr-gpu*, *xcorr2-cl* on Q RTX 6000 hardware configuration.

Q RTX 6000	xcorr (KISS)	xcorr-gpu		xcorr2-cl	
	Secs	Secs	Speed-Up	Secs	Speed-Up
ALOS1-std	154.501	3.701	41.746	4.394	35.162
ALOS1-ERSDAC	155.801	2.427	64.195	3.669	42.464
ALOS1-iono	161.377	3.007	53.667		
ALOS2-L1.1	154.458	3.076	50.214		
ALOS2-SCAN	292.804	6.152	47.595	9.632	30.399
ENVI	155.429	2.349	66.168	3.651	42.572
ERS	155.089	2.414	64.246	3.849	40.293
CSK	157.835	5.676	27.807	6.366	24.793
RS2	153.147	2.017	75.928	3.565	42.958
TSX	156.963	4.562	34.407		

**Table 6:** Execution times for *xcorr* using FFTW and Brenner FFT on GTX 1050 Ti hardware configuration. Figures are expressed in seconds, and the *sys* column presents the fraction of the total spent in Supervisor mode, also in seconds.

GTX 1050 Ti	xcorr (FFTW)		xcorr (Brenner)	
	Total	sys	Total	sys
ALOS1-std	254.346	38.941	82.857	0.288
ALOS1-ERSDAC	253.54	38.969	83.219	0.145
ALOS1-iono	254.172	38.407	83.215	0.168
ALOS2-L1.1	255.199	38.490	83.479	0.197
ALOS2-SCAN	1905.077	48.473	161.231	0.6
ENVI	254.147	38.349	83.184	0.168
ERS	254.104	39.050	83.555	0.216
CSK	254.59	39.368	86.213	0.528
RS2	251.816	38.297	82.89	0.112
TSX	255.77	38.135	85.507	0.325

**Table 7:** Execution times and Speed-Up for *xcorr-gpu*, *xcorr2-cl* on GTX 1050 Ti hardware configuration. Figures in *total* and *sys* columns are expressed in seconds, where sys represent the time spent in Supervisor mode. *S-Up* columns represent speed-ups expressed with respect to *xcorr* execution times using both FFTW and Brenner FFT.

GTX 1050 Ti	xcorr-gpu				xcorr2-cl			
	Total	sys	S-Up FFTW	S-Up Brenner	Total	sys	S-Up FFTW	S-Up Brenner
ALOS1-std	4.259	0.414	59.720	19.455	7.011	0.313	36.278	11.818
ALOS1-ERSDAC	3.308	0.334	76.644	25.157	5.133	0.225	49.394	16.213
ALOS1-iono	3.271	0.381	77.705	25.440				
ALOS2-L1.1	3.854	0.352	66.217	21.660				
ALOS2-SCAN	9.367	0.901	203.382	17.213	16.326	0.673	116.690	9.876
ENVI	3.179	0.351	79.946	26.167	4.961	0.217	51.229	16.768
ERS	3.367	0.329	75.469	24.816	5.272	0.229	48.199	15.849
CSK	6.996	0.908	36.391	12.323	13.274	2.116	19.180	6.495
RS2	2.936	0.26	85.768	28.232	4.375	0.213	57.558	18.946
TSX	5.681	0.709	45.022	15.051				

**Table 8:** Execution times for *xcorr-gpu* with power mode set on 5 Watts and 10 Watts on Jetson Nano hardware configuration. Figures are expressed in seconds, and the *sys* column presents the fraction of the total spent in Supervisor mode, also in seconds.

Jetson Nano	xcorr-gpu 10 W		xcorr-gpu 5 W	
	Total	sys	Total	sys
ALOS1-std	48.912	14.332	56.569	18.18
ALOS1-ERSDAC	30.068	11.996	40.091	14.856
ALOS1-iono	30.206	12.176	41.937	15.756
ALOS2-L1.1	34.412	12.316	47.454	15.492
ALOS2-SCAN	110.219	37.368	132.804	51.24
ENVI	29.159	11.828	38.612	14.568
ERS	30.671	12.236	40.372	14.728
CSK	77.725	17.076	92.772	21.176
RS2	26.936	11.86	35.33	14.568
TSX	65.208	15.624	76.576	20.668

## Data Availability

The source code of the software presented in this study is openly available in GitHub and Zenodo at https://doi.org/10.5281/zenodo.5142645 (Last accessed on 31 August 2021).
